# Differential responses of pulmonary vascular cells from PAH patients and controls to TNFα and the effect of the BET inhibitor JQ1

**DOI:** 10.1186/s12931-023-02499-y

**Published:** 2023-07-29

**Authors:** Sharon Mumby, Frederic Perros, Julien Grynblat, Gregoire Manaud, Alberto Papi, Paolo Casolari, Gaetano Caramori, Marc Humbert, S. John Wort, Ian M. Adcock

**Affiliations:** 1 Respiratory Science, NHLI, Imperial College London, London, UK; 2 Inserm UMR-S 999, Hôpital Marie Lannelongue, Groupe Hospitalier Paris Saint Joseph, Université Paris-Saclay, Le Plessis-Robinson, France; 3 CarMeN Laboratory, INSERM U1060, INRAE U1397, Université Claude Bernard Lyon1, Pierre-Bénite, France; 4 Interdepartmental Study Center for Inflammatory and Smoke-Related Airway Diseases, Cardiorespiratory and Internal Medicine Section, University of Ferrara, Ferrara, Italy; 5 Pneumologia, Dipartimento di Scienze Biomediche, Odontoiatriche e Delle Immagini Morfologiche e Funzionali (BIOMORF), Università Degli Studi di Messina, Messina, Italy; 6 Department of Respiratory and Intensive Care Medicine, AP-HP, Hôpital Bicêtre, Pulmonary Hypertension National Referral Center, Université Paris-Saclay, Le Kremlin-Bicêtre, France; 7 National Pulmonary Hypertension Service, Royal Brompton Hospital, London, UK

**Keywords:** Bromodomain and extra‐terminal proteins, Human pulmonary microvascular endothelial cells, Human pulmonary artery smooth muscle cells, Inflammation, Pulmonary arterial hypertension

## Abstract

**Background:**

Pulmonary arterial hypertension (PAH) encompasses a group of diseases characterized by raised pulmonary vascular resistance, resulting from vascular remodelling and inflammation. Bromodomain and extra-terminal (BET) proteins are required for the expression of a subset of NF-κB-induced inflammatory genes which can be inhibited by the BET mimic JQ1+. We hypothesised that JQ+ would supress TNFα-driven inflammatory responses in human pulmonary vascular cells from PAH patients.

**Methods:**

Immunohistochemical staining of human peripheral lung tissue (N = 14 PAH and N = 12 non-PAH) was performed for the BET proteins BRD2 and 4*.* Human pulmonary microvascular endothelial cells (HPMEC) and pulmonary artery smooth muscle cells (HPASMC) from PAH patients (N = 4) and non-PAH controls (N = 4) were stimulated with TNFα in presence or absence of JQ1+ or its inactive isomer JQ1–. IL-6 and -8 mRNA was measured by RT-qPCR and protein levels by ELISA. Chromatin immunoprecipitation analysis was performed using EZ-ChIP™ and NF-κB p65 activation determined using a TransAm kit. MTT assay was used to measure cell viability.

**Results:**

Nuclear staining of BRD2 and BRD4 was significantly (p < 0.0001) increased in the lung vascular endothelial and smooth muscle cells from PAH patients compared to controls with normal lung function. TNFα-driven IL-6 release from both HPMECs and HPASMCs was greater in PAH cells than control cells. Levels of CXCL8/IL-8 protein release was higher in PAH HPASMCs than in control cells with similar release observed in HPMECs. TNFα-induced recruitment of activated NF-κB p65 to the IL-6 and CXCL8/IL-8 promoters were similar in both cell types and between subject groups. JQ1+ suppressed TNFα-induced IL-6 and CXCL8/IL-8 release and mRNA expression to a comparable extent in control and PAH HPMECs and HPASMCs. JQ1 had a greater efficacy on IL-6 release in HPMEC and on CXCL8/IL-8 release in HPASMC.

**Conclusion:**

BET inhibition decreases TNFα driven inflammation in primary pulmonary vascular cells. The anti-inflammatory actions of JQ1 suggests distinct cell-specific regulatory control of these genes. BET proteins could be a target for future therapies for PAH.

**Supplementary Information:**

The online version contains supplementary material available at 10.1186/s12931-023-02499-y.

## Introduction

Pulmonary arterial hypertension (PAH) is a rare and incurable progressive disease of the lung vasculature which leads to right heart failure and death. It encompasses a group of diseases characterized by raised pulmonary vascular resistance, resulting from vascular remodelling in the pre-capillary resistance arterioles [1, 2]. Many factors contribute to the vasoconstriction and vascular remodelling, but despite therapeutic advances for PAH the 3-year survival is only ~ 70% [2–4]. Treatment with pulmonary vasodilators, such as endothelin receptor antagonists, prostacyclin analogues and phosphodiesterase type V inhibitors have improved both morbidity and mortality but are not a cure [2, 5]. It is important, therefore, to understand the mechanisms of vascular remodelling in PAH and to determine novel therapies targeting these abnormalities.

Vascular remodelling is common to all types of PAH and in early stages of the disease there is a significant increase in inflammation and oxidative stress causing cell damage and programmed cell death [6, 7]. In contrast, increased proliferation is a hallmark of later disease [8]. Markers of increased inflammation, such as nuclear factor kappa-light-chain-enhancer of activated B cells (NF-κB) activation, is seen in pulmonary vascular endothelial cells in patients with idiopathic PAH (IPAH) [9]. Endothelial cells, in vitro, produce cytokines that are also found circulating in the plasma of patients with IPAH and correlate with worse survival [10].

Epigenetic changes such as histone acetylation regulate inflammatory gene expression and cell proliferation, migration and survival [11, 12]. The histone acetylation/deacetylation balance is maintained by histone acetyltransferases (HATs) and histone deacetylases (HDACs) which control chromatin structure and the accessibility of transcription factors for their target genes [13]. Histone acetylation, is linked to heightened inflammatory gene expression [14, 15]. Bromodomains (BRDs) are conserved structures that read acetylated lysine’s to interpret the histone acetylation topography. BRD-containing bromodomain and extra-terminal (BET) proteins co-ordinate the regulation of genes involved in cell proliferation, apoptosis, and inflammation [16, 17].

The expression of BET proteins, specifically BRD4, is increased in PAH lung tissue, distal pulmonary arteries (PAs) and right ventricle (RV) compared to control tissues [18, 19]. Overexpression was also demonstrated in isolated PAH pulmonary artery smooth muscle cells (PASMCs) compared to control cells [18]. However, both BRD2 and 4 regulate inflammatory gene expression in several murine and human cell types [20–22] and molecular mimics such as JQ1+, which block binding to acetylated histones, attenuate cell proliferation and differentiation in vivo and in cell lines [23–25]. I-BET151, a structurally similar BET mimic, reduced inflammation in vivo and in vitro following LPS challenge [23]. In the Sugen/hypoxia rat model, BRD4 inhibition using JQ1 or siBRD4 in vivo reversed established PAH and decreased proliferation, increased apoptosis and restored mitochondrial membrane potential in PAH-PASMCs, suggesting that BRD4 upregulation may be pathologically associated with PAH [18].

The clinically available BET inhibitor, RVX208 (Apabetalone) showed promise in preclinical trials [26], where it reversed PA remodelling in various PAH rat models, potentially via modulation of proinflammatory, proproliferative and prosurvival pathways. Apabetalone has undergone phase I-III cardiovascular trials where it failed to show a significant effect on cardiovascular death but did show promise on secondary outcomes and was well tolerated [27]. A recent pilot study, clinical trial (NCT 03655704) was carried out to test the feasibility of Apabetalone for a future early-stage trial to evaluate inhibition of BRD in PAH [28]. This single-arm open-label study reported that apabetalone was feasible and that further studies are required to confirm the efficacy signal but it may be associated with beneficial effects when added to current PAH therapies.

We hypothesised that BET mimics will effectively suppress inflammation-driven functions of human pulmonary artery smooth muscle (HPASMC) and pulmonary microvascular endothelial cells (HPMEC). We investigated the effect of the BET mimic JQ1+ on the regulation of TNFα-driven inflammatory responses in HPASMCs and HPMECs from PAH patients and non-PAH subjects and determined the expression of BRD2 and 4 in vascular cells from PAH patients and control lung tissue.

## Methods

### Tissue collection and cell isolation

Demographics for the donors used for cell culture have previously been described [29]. See Table 1 for the donor demographics for the peripheral lung tissue sections.

**Table 1:** Donor demographics for the immunohistochemical study of BRDs in peripheral lung tissue. Data are expressed as mean ± SEM

Subjects	Number	Age (years)	Sex
Non-PAH controls	12	68.2 ± 2.4	9 M/3F
PAH	14	38.5 ± 2.9	7 M/7F

#### Cell culture and treatment

Both cell types were cultured until 70% confluent at 37 °C and 5% CO_2_. 24 h prior to experiments, media was changed to EGM2 containing 0.1% FCS for HPMEC and 0.1% BSA for HPASMC before being returned to media containing 5% FCS in the presence or absence of the BET mimic JQ1+ or its inactive enantiomer JQ1– for 2 h prior to the addition of TNFα for varying amounts of time depending on the experiment.

#### Measurement of mRNA transcripts

Total RNA was isolated using RNeasy mini kit (Qiagen Ltd, Crawley, UK). Single stranded cDNA was synthesized from quantified RNA by reverse transcription using a high-capacity cDNA kit (Applied Biosystems), following the manufacturer's instructions. QPCR was performed in a Rotor-Gene 6000 PCR machine (Corbett Research, Cambridge, UK) using a QuantiTect SYBR Green PCR kit and Quantitect primers (Qiagen). PCR data for each gene was normalised to a housekeeping gene, β-actin and represented as fold change respective to the t = 0 or 4 h time point, using the delta-delta CT (2-^∆∆CT^) method [30].

#### ELISAs

IL-6 and CXCL8 levels in culture supernatants were analysed by sandwich ELISA (BioTechne, Oxford, UK).

#### NF-κB p65 activation assay

Cells were collected, and nuclear extracts prepared using a nuclear extract kit (Active Motif Europe, Belgium), according to the manufacturer’s instructions. Protein concentration was determined by the Bradford assay (Bio-Rad Laboratories Ltd, Hemel Hempstead, UK). NF-κB p65 activation was measured using TransAM NF-κB p65 activation kit (Active Motif) following the manufacturer’s instructions.

#### Chromatin immunoprecipitation (ChIP) assay

ChIP assay was carried out using the EZ-ChIP™ kit (Millipore, Watford, UK) according to the manufacture’s protocol as previously described [29].

#### Immunoperoxidase staining of FF/PE human peripheral lung

Sections were dewaxed and rehydrated as previously described [9], followed by heat-mediated antigenic retrieval using a pH 6.0 citrate buffer (BRD2) or a pH 8.0 EDTA buffer (BRD4). Endogenous peroxidase activity was quenched by incubation of the slides in 3% hydrogen peroxide for 30 min; cell membranes were permeabilised by 0.1% saponin in PBS, and non-specific labelling blocked by 5% normal horse serum (BRD2) or 5% normal goat serum (BRD4) in PBS for 20 min, at room temperature (as for all the following steps). After washing in PBS, sections were incubated for 1 h with mouse anti-human BRD2 (sc-393720; Santa Cruz Biotechnology) at a 1∶2900 dilution (0.069 µg/ml) or with rabbit anti-human BRD4 (ab128874; Abcam) at a 1∶300 dilution (1.577 µg/ml). For negative control slides, normal mouse or normal rabbit non-specific immunoglobulins (Santa Cruz Biotechnology) were used at the same protein concentration as the primary antibodies. After repeated PBS washing, sections were incubated with anti-mouse or anti-rabbit biotinylated antibody (Vector ABC Kit, Vector Laboratories) for 30 min. Sections were then incubated with ABC reagent (Vector ABC Kit, Vector Laboratories) for 30 min and stained with chromogen-fast diaminobenzidine (DAB) for 1–5 min. Finally, the slides were counterstained in haematoxylin and mounted on permanent mounting medium.

### Statistical analysis

Data are represented as mean ± SEM. Data were analysed by Student’s *t* test for two groups of data, by one-way ANOVA/Dunn’s multiple comparison test for more than two data sets using GraphPad Prism (La Jolla, CA, USA). Differences were considered significant for p ≤ 0.05.

## Results

The authors would like to highlight that the HPMEC data from non-PAH controls presented in this manuscript has previously been published [31] but is shown here in this paper for comparison to the data in PAH.

### BRD2 and 4 expression in vascular cells from PAH patients and controls

**Fig. 1:**
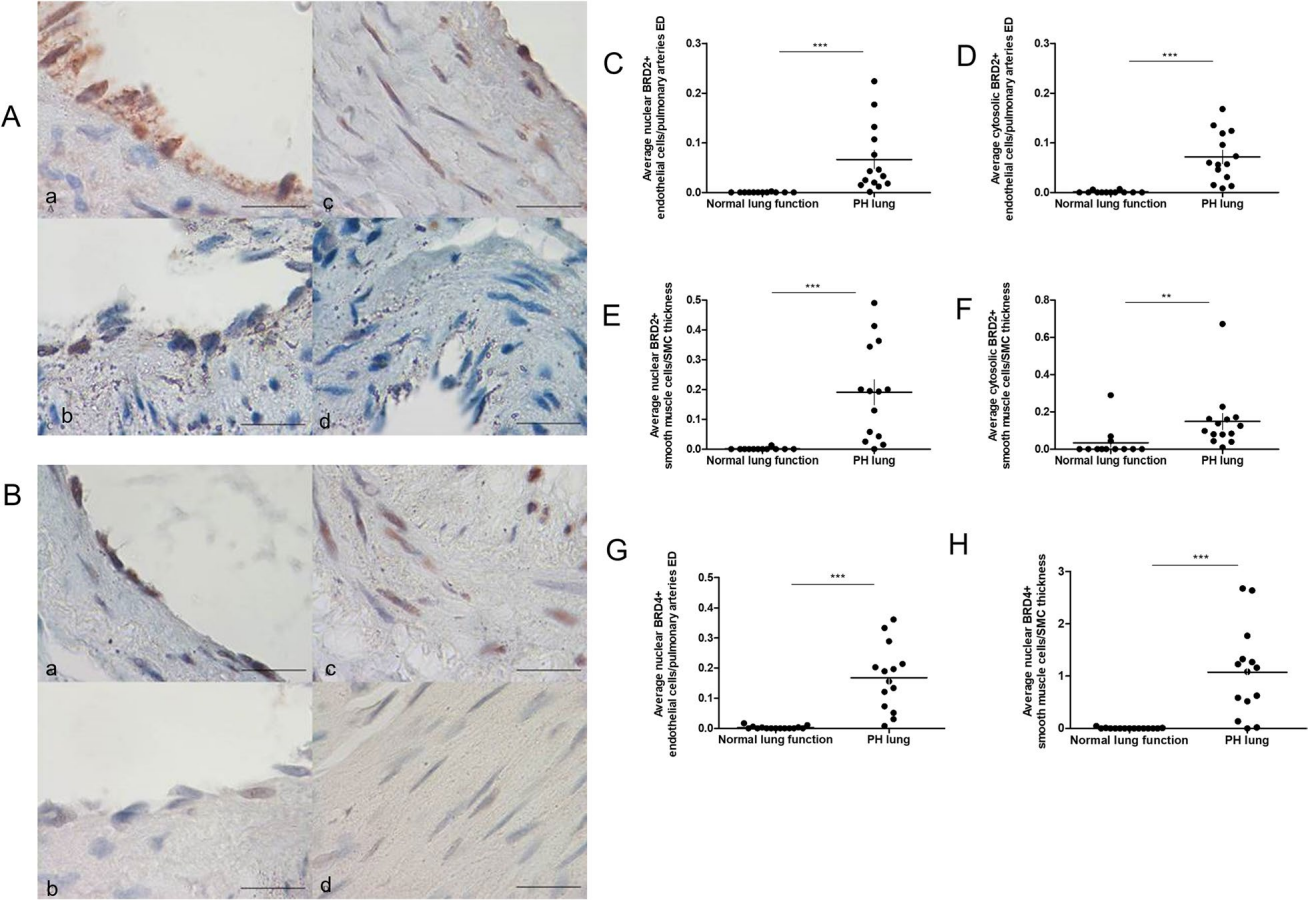
BRD2 and 4 protein expression in vascular cells from PAH patients and controls. Immunohistochemistry was performed on lung tissue sections from control (n = 12) and PAH (n = 14) patients to determine levels of BRD2 and 4 protein expression. **A** BRD2 expression in pulmonary endothelial cells from PAH patients (**a**) and controls (**b**) and smooth muscle cells from PAH patients (**c**) and controls (**d**). **B** BRD4 expression in pulmonary endothelial cells from PAH patients (**a**) and controls (**b**) and smooth muscle cells from PAH patients (**c**) and controls (**d**). × 100 magnification, bar = 20 μm. Slides were scored and average BRD2 expression in the nucleus and cytosol of pulmonary endothelial cells **C** and **D** and smooth muscle cells **E** and **F** plotted. BRD4 expression in the nucleus of pulmonary endothelial cells **G** and smooth muscle cells **H**. Data are shown as scatter plots with mean ± SEM. Statistical comparisons were made using non-parametric t-test **p < 0.001, ***p < 0.0001

Representative images of BRD2 and BRD4 immunohistochemical staining in HPMECs and HPASMCs from PAH and control subjects are shown in Fig. 1A and B respectively. Nuclear and cytosolic levels of BRD2 were significantly (p < 0.0001) increased in the endothelium (Fig. 1C and D respectively) and smooth muscle cells (Fig. 1E and F respectively) in lung tissue from PAH patients compared to controls with normal lung function. There was a significant (p < 0.001) increase in nuclear BRD4 expression in both cell types in PAH patients compared to controls (Fig. 1G and H) but no increase in cytosolic BRD4 was seen.

### TNFα increases IL-6 and CXCL8 protein levels in HPMEC

Concentration and time course experiments were performed with TNFα to elucidate the optimum conditions for release of IL-6 and CXCL8 protein from pulmonary endothelial cells. Incubation of control and PAH HPMEC with TNFα (0-100 ng/ml) resulted in a concentration-dependent increase in the release of IL-6 at 6, 16 and 24 h (Additional file 1: Fig. S1A and B). In response to TNFα endothelial cells from PAH patients released approximately four times more IL-6 at 24 h (1.63 ± 0.38 v 0.45 ± 0.22 ng/ml). Similar time- and concentration-dependent increases in CXCL8 release were seen in TNFα-treated (0-100 ng/ml) cells, except that control and PAH cells released similar levels of CXCL8 in response to TNFα (Additional file 1: Fig. S1C and D). TNFα (10 ng/ml) was selected for future experiments due to loss of cell viability with 100 ng/ml at 24 h (data not shown).

### TNFα increases IL-6 and IL-8 mRNA levels in HPMEC

TNFα (10 ng/ml) caused a rapid and significant (p ≤ 0.001) induction of IL-6 mRNA at 2 h in both control and PAH HPMEC. IL-6 mRNA levels continued to increase in control cells and plateaued at around 8 h, however the levels declined in PAH cells after 2 h but a secondary peak was observed at 24 h (Additional file 2: Fig. S2A and B). TNFα-induced IL-6 mRNA was significantly higher in control endothelial cells compared to that in PAH patients.

There was a large and highly significant (p < 0.0001) increase in IL-8 mRNA at 2 h in response to TNFα in both control and PAH endothelial cells. With control cells increasing IL-8 mRNA levels 150 times and PAH cells over 30 times compared to untreated levels (Additional file 2: Fig. S2C and D). In control cells, IL-8 mRNA levels gradually declined between 4-24 h whereas in PAH cells they increased but still remained lower than levels seen in control cells. TNFα treatment resulted in a greater induction of IL-8 mRNA in control HPMEC compared to PAH cells. In all subsequent mRNA experiments cells were incubated with TNFα for 4 h.

### Effect of JQ1 on TNFα stimulated IL-6 and CXCL8 protein levels in HPMEC form PAH patients and controls

HPMEC were pre-incubated for 2 h with different concentrations (0–1000 nM) of JQ1+ or the inactive compound JQ1– prior to treatment with 10 ng/ml TNFα for 24 h.

**Fig. 2:**
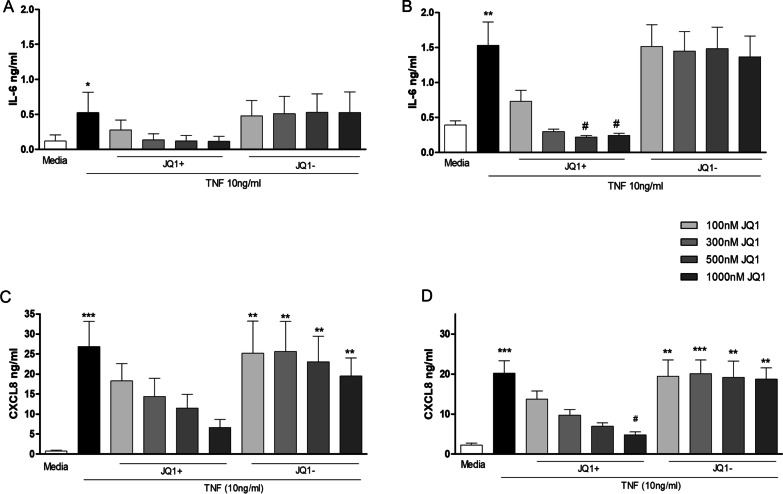
Effect of JQ1 on TNFα stimulated IL-6 and CXCL8 levels in HPMEC. Control (lefthand panels) and PAH (righthand panels) HPMECs were pre-incubated with JQ1 + or JQ1- for 2 h prior to the addition of TNFα (10 ng/ml) for 24 h. Culture media was collected and levels of IL-6 **A** and **B** and CXCL8 **C** and **D** determined by ELISA. Data are shown as mean ± SEM of N = 8 independent experiments (4 Donors at 2 different passages). Statistical comparisons were made using Kruskal–Wallis one-way ANOVA with Dunns post-test. *p < 0.05, **p < 0.01, ***p < 0.001 when compared to media control and ^#^p < 0.05 when compared to TNFα.

JQ1+ , but not JQ1–, decreased TNFα-stimulated release of IL-6 from control and PAH endothelial cells (Fig. 2A and B). The inhibitory effects of JQ1 + only reached significance (p < 0.05) at concentrations of 500 and 1000 nM in PAH-HPMEC. The IC_50_ for JQ1 + on TNFα-stimulated IL-6 release in HPMEC was 93 ± 20 nM in controls and 108 ± 14 nM in PAH derived cells.

JQ1 + showed a concentration-dependent decrease in CXCL8 release from TNFα-stimulated control and PAH HPMEC, reaching significance with 1000 nM JQ1 + in PAH cells (Fig. 2C and D). JQ1– had no effect on TNFα stimulated CXCL8 release. The IC_50_ for JQ1+ suppression of TNFα-induced CXCL8 release was 3 times higher than that required for TNFα-stimulated IL-6 release in both control (308 ± 70 nM) and PAH cells (313 ± 24 nM). For further experiments, pre-treatment of cells for 2 h with JQ1 ± (1000 nM) was used as no detectable loss in cell viability was seen.

### Effect of JQ1 on TNFα stimulated induction of IL-6 and IL-8 mRNA levels in HPMEC

**Fig. 3:**
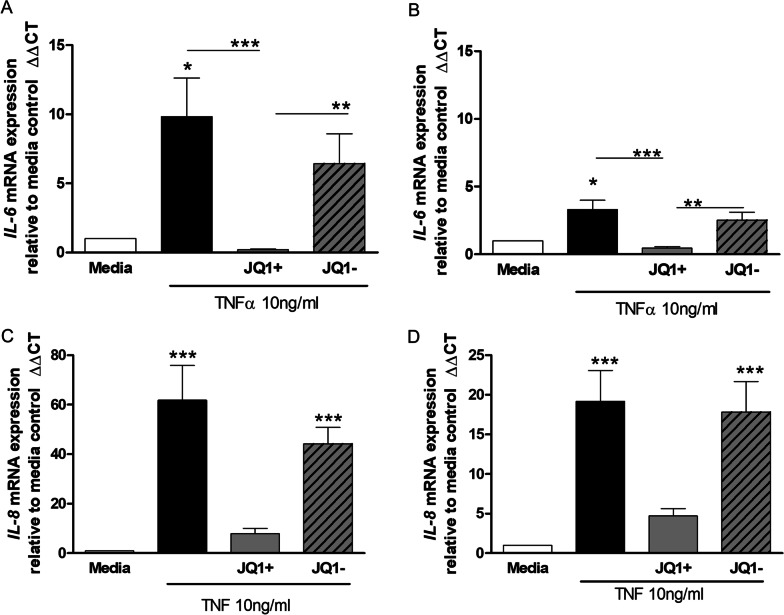
Effect of JQ1 on TNFα stimulated induction of IL-6 and IL-8 mRNA levels in HPMEC. HPMECs from control (lefthand panels) and PAH (righthand panels) patients were pre-incubated with JQ1 ± (1000 nM) for 2 h prior to the addition of TNFα (10 ng/ml) for 4 h. Cells were collected, RNA extracted, after which cDNA synthesis and RT-PCR was performed for IL-6 (**A** and **B**) and IL-8 (**C** and **D)**. Data are shown as mean ± SEM of N = 8 (4 Donors at 2 different passages) experiments. Statistical comparisons were made using Kruskal-Wallis one-way ANOVA with Dunns post-test. *p < 0.05, **p < 0.01, ***p < 0.001 when comparing to media control or as indicated by the line

JQ1+, but not JQ1–, significantly (p < 0.001) inhibited TNFα-stimulated IL-6 mRNA induction in HPMEC from both controls and PAH patients (Fig. 3A and B). JQ1 + had a greater effect in control cells compared to PAH-derived cells even though control cells gave a three times greater induction of IL-6 mRNA.

JQ1+ , but not JQ1–, also decreased TNFα-stimulated IL-8 mRNA induction in HPMEC from control and PAH patients by 80–90% but this did not reach significance for either group (Fig. 3C and D). Again, induction of mRNA levels by TNFα was three times higher in control cells and JQ1+ had a greater effect on TNFα-stimulated IL-8 induction in these cells compared to disease.

### Effect of JQ1 on TNF stimulated enrichment of NF-κB p65 and BRD4 to -κB binding sites on IL-6 and IL-8 promotors

HPMEC were pre-incubated with JQ1 ± (1000 nM) for 2 h prior to treatment with TNFα (10 ng/ml) for up to 4 h for TransAm analysis and for 3 h in ChIP assays. JQ1+ had no effect on TNFα-stimulated NF-κB p65 DNA binding in HPMECs from control or PAH subjects measured by TransAm over the time-course studied (30–240 min, data not shown).

**Fig. 4:**
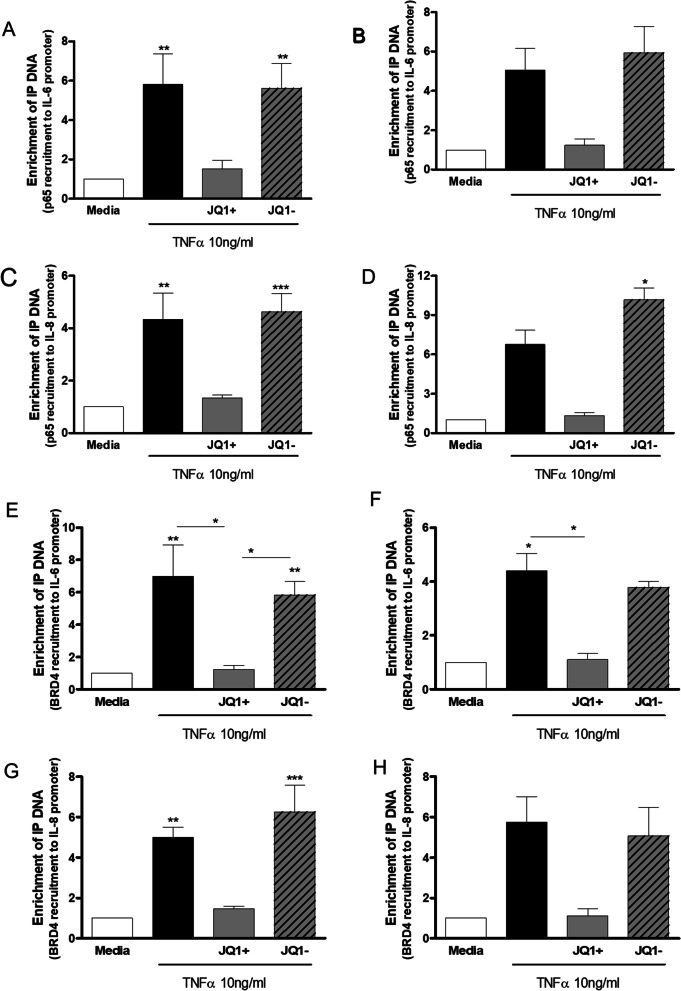
Effect of JQ1 on TNF stimulated enrichment of NF-κB p65 and BRD4 to -κB binding sites on IL-6 and IL-8 promotors in HPMEC. HPMEC from control (lefthand panels) and PAH (righthand panels) patients were pre-incubated with JQ1 ± (1000 nM) for 2 h prior to treatment with TNFα (10 ng/ml) for 3 h. Cells were harvested and ChIP analysis performed to determine enrichment of NF-κB p65 to κB binding sites on IL-6 **A** and **B** and IL-8 **C** and **D** promotors. Additionally, enrichment of BRD4 to -κB binding sites on IL-6 **E** and **F** and IL-8 **G** and **H** promotors was determined. Data are shown as mean ± SEM of N = 8 (4 Donors at 2 different passages) experiments. Statistical comparisons were made using Kruskal-Wallis one-way ANOVA with Dunns post-test. *p < 0.05, **p < 0.01, ***p < 0.001 when comparing to media control or as indicated by the line

ChIP analysis showed that TNFα enhanced NF-κB p65 recruitment to κB binding sites in the IL-6 and IL-8 promotor regions to a similar extent in HPMEC from controls and PAH patients (Fig. 4). This increase was significant (p < 0.01) in controls but failed to reach significance in cells from patients with PAH. Pre-incubation with JQ1 + resulted in a decrease in recruitment of NF-κB p65 to the κB binding site on the IL-6 (Fig. 4A and B) and IL-8 (Fig. 4C and D) promotor regions to near basal levels in control and PAH HPMEC.

TNFα stimulation of HPMEC from control and PAH patients resulted in an increase in the recruitment of BRD4 to the κB binding site on the IL-6 and IL-8 promotor regions (Fig. 4). This increase was significant (p < 0.05) for both controls and PAH cells for the IL-6 promotor (Fig. 4E and F) but only significant (p < 0.01) for the control cells for recruitment of BRD4 to IL-8 promotor (Fig. 4G and H).

Pre-treatment with JQ1 + significantly (p < 0.05) reduced TNFα-induced BRD4 recruitment to the IL-6 κB site (Fig. 4E and F). JQ1 + also decreased TNFα-stimulated recruitment of BRD4 to the IL-8 κB site to near basal levels but this failed to reach statistical significance (Fig. 4G and H). JQ1- had no effect on the binding of NF-κB p65 or BRD4 to the IL-6 or IL-8 promoter κB binding sites.

### TNFα increases IL-6 and CXCL8 protein levels in HPASMC

As with HPMECs, concentration- and time-course experiments were performed with TNFα to elucidate the optimum conditions for release of IL-6 and CXCL8 protein from HPASMCs. Incubation of control and PAH HPASMC with TNFα (0-100 ng/ml) resulted in a time- and concentration-dependent increase in IL-6 release (Additional file 3: Fig. S3A and B). Significant (p < 0.05) increases in IL-6 were seen with TNFα concentrations > 1 ng/ml at 16 and 24 h, with cells from PAH patients releasing more IL-6 then controls in response to TNFα. Both control and PAH HPASMCs released 10 times more IL-6 in response to TNFα compared to HPMECs.

Similar time- and concentration-dependent increases in CXCL8 release were seen in TNFα (0-100 ng/ml)-treated cells, with PAH cells releasing almost double the amount of CXCL8 in response to TNFα compared to healthy cells (Additional file 3: Fig. S3C and D). Healthy HPASMC and HPMEC released similar levels of CXCL8, but PAH HPASMC released double the amount of CXCL8 compared to HPMEC in response to TNFα. In all subsequent experiments, 10 ng/ml TNFα was used due a drop in cell viability with 100 ng/ml at 24 h (data not shown).

### TNFα increases IL-6 and IL-8 mRNA levels in HPASMC

TNFα (10 ng/ml) caused a large and significant (p ≤ 0.01) induction of IL-6 mRNA at 2 h in both control and PAH HPASMC (Additional file 4: Fig. S4A and B). IL-6 mRNA levels continued to increase in control cells up to 8 h and then declined but were still significantly (p < 0.01) increased compared to untreated cells at 24 h. In PAH cells after the initial rapid increase in IL-6 mRNA at 2 h the levels slowly decreased but remained significantly increased compared to untreated cells. TNFα caused a threefold greater induction of IL-6 mRNA in control HPASMC compared to PAH cells. A greater induction of IL-6 mRNA in response to TNFα was seen in HPASMCs compared to HPMECs (threefold in control and twofold in PAH cells).

Stimulation of HPASMC with TNFα resulted in a significant (p < 0.01) induction of IL-8 mRNA at 2 h which continued to increase to around 8 h where levels were some 6000 times higher than basal levels in control cells and 700 times higher in PAH HPASMC. At 24 h post TNFα treatment IL-8 mRNA levels had declined but were still significantly (p < 0.05) higher than untreated cells (Additional file 4: Fig. S4C and D). TNFα caused a tenfold greater induction of IL-8 mRNA in control HPASMC compared to PAH cells. In comparison to HPMECs treated with TNFα, HPASMC showed a far greater induction of IL-8 mRNA in control and PAH cells (30-fold in control and 20-fold in PAH cells).

### Effect of JQ1 + on TNFα stimulated IL-6 and CXCL8 protein levels in HPASMC

**Fig. 5:**
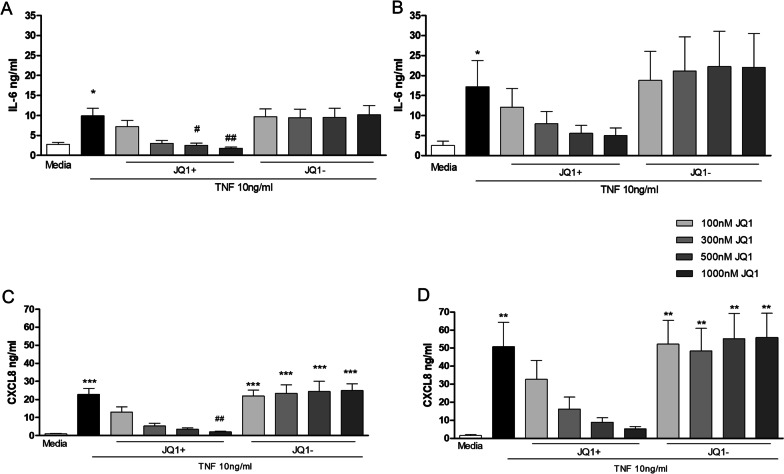
Effect of JQ1 on TNFα stimulated IL-6 and CXCL8 levels in HPASMC. Control (lefthand panels) and PAH (righthand panels) HPASMCs were pre-incubated with JQ1 + or JQ1- for 2 h prior to the addition of TNFα (10 ng/ml) for 24 h. Culture media was collected and levels of IL-6 **A** and **B** and CXCL8 **C** and **D** determined by ELISA. Data are shown as mean ± SEM of N = 8 experiments (4 Donors at 2 different passages). Statistical comparisons were made using Kruskal-Wallis one-way ANOVA with Dunns post-test. *p < 0.05, **p < 0.01, ***p < 0.001 when compared to Media control and ^#^p < 0.05, ^##^p < 0.01 when compared to TNFα

As with HPMECs, HPASMC were pre-incubated for 2 h with JQ1+ or JQ1– (0–1000 nM) prior to treatment with 10 ng/ml TNFα for 24 h. JQ1+ , but not JQ1–, decreased TNFα-stimulated IL-6 release from control and PAH cells (Fig. 5A and B). The inhibitory effects of JQ1 + only reached significance (p < 0.05) at 500 and 1000 nM in control-HPASMC. The IC_50_ for JQ1 + in HPASMC was 181 ± 27 nM in controls and 248 ± 50 nM in PAH-derived cells. The IC_50_ for JQ1 + in HPASMC was double that required for HPMEC.

JQ1 + showed a concentration-dependent decrease in CXCL8 release from TNFα-stimulated control and PAH HPASMC, reaching significance (p < 0.01) with 1000 nM JQ1 + in control cells (Fig. 5C and D). The JQ1 + IC_50_ in control cells was 115 ± 24 nM and in PAH cells was 140 ± 40 nM. These IC_50_ values were threefold lower than for HPMECs. JQ1– had no effect on TNFα stimulated CXCL8 release. For further experiments JQ1 ± was used at 1000 nM as no detectable loss in cell viability was seen.

### Effect of JQ1 + on TNFα stimulated IL-6 and IL-8 mRNA levels

**Fig. 6:**
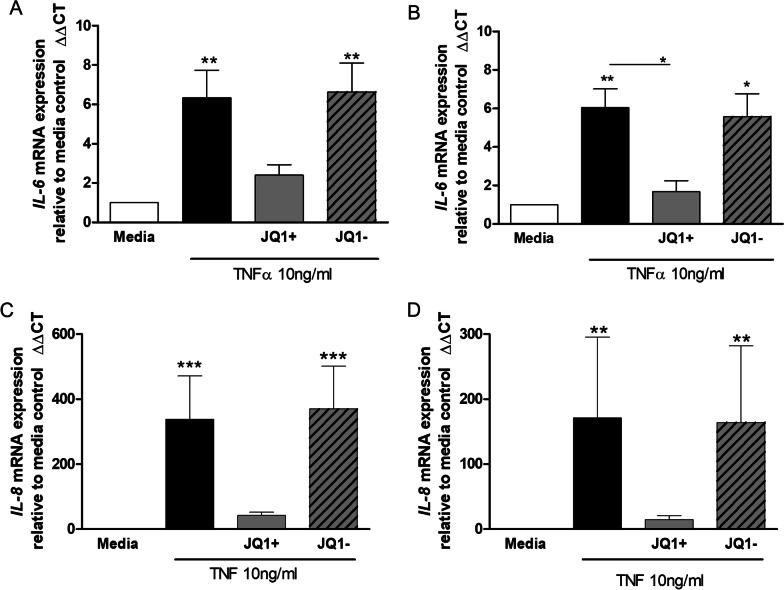
Effect of JQ1 on TNFα stimulated induction of IL-6 and IL-8 mRNA levels in HPASMC. HPASMCs from control (lefthand panels) and PAH (righthand panels) patients were pre-incubated with JQ1 ± (1000 nM) for 2 h prior to the addition of TNFα (10 ng/ml) for 4 h. Cells were collected, RNA extracted, after which cDNA synthesis and RT-PCR was performed for IL-6 **A** and **B** and IL-8 **C** and **D**. Data are shown as mean ± SEM of N = 8 (4 Donors at 2 different passages) independent experiments. Statistical comparisons were made using Kruskal-Wallis one-way ANOVA with Dunns post-test. *p < 0.05, **p < 0.01, ***p < 0.001 when comparing to media control or as indicated by the line

JQ1+ decreased TNFα-stimulated IL-6 mRNA induction in HPASMC reaching significance in PAH cells (Fig. 6A and B). JQ1- had no effect on TNFα stimulated IL-6 mRNA induction.

JQ1+, but not JQ1-, also decreased the large increase in TNFα-stimulated IL-8 mRNA induction in HPASMC from controls and PAH patients by 80–90% but this did not reach statistical significance (Fig. 6C and D).

### Effect of JQ1 + on TNFα-stimulated enrichment of NF-κB p65 and BRD4 to -κB binding sites on IL-6 and IL-8 promotors

As with HPMECs, TransAm analysis showed that JQ1 ± had no effect on TNFα-stimulated NF-κB p65 nuclear translocation in HPASMC from control and PAH patients over 0–4 h (data not shown).

**Fig. 7:**
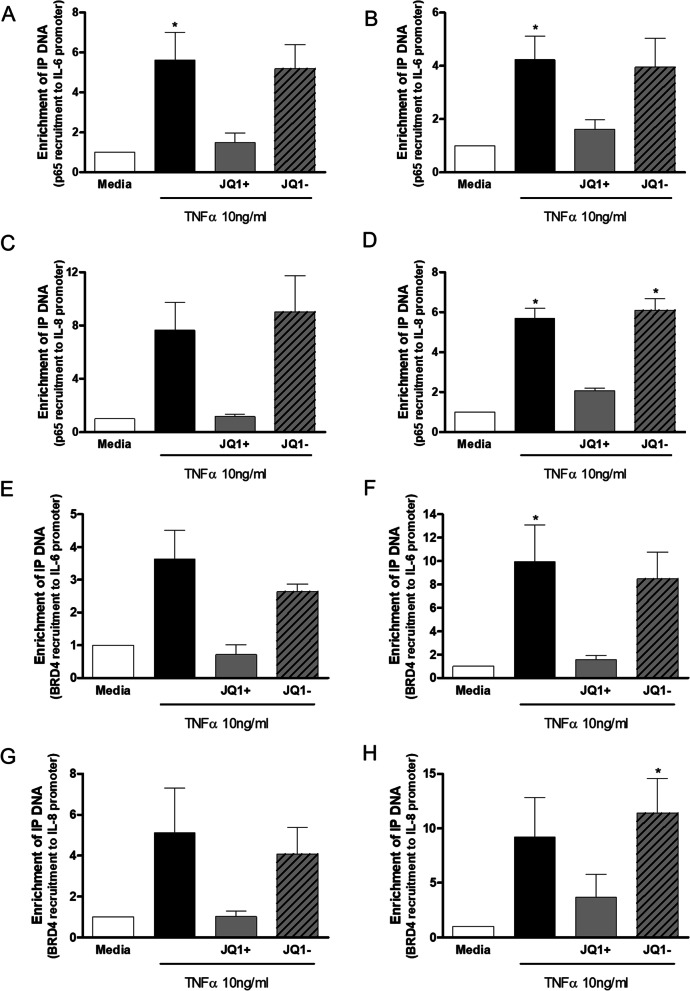
Effect of JQ1 on TNF stimulated enrichment of NF-κB p65 and BRD4 to -κB binding sites on IL-6 and IL-8 promotors in HPASMC. HASMCs from control (lefthand panels) and PAH (righthand panels) patients were pre-incubated with JQ1 ± (1000 nM) for 2 h prior to treatment with TNFα (10 ng/ml) for 3 h. Cells were harvested and ChIP analysis performed to determine enrichment of NF-κB p65 to κB binding sites on IL-6 **A** and **B** and IL-8 **C** and **D** promotors. Additionally, enrichment of BRD4 to -κB binding sites on IL-6 **E and F** and IL-8 **G** and **H** promotors was determined. Data are shown as mean ± SEM of N = 8 (4 Donors at 2 different passages) experiments. Statistical comparisons were made using Kruskal-Wallis one-way ANOVA with Dunns post-test. *p < 0.05, when comparing to media control

In HPASMC from controls and PAH patients TNFα stimulation resulted in a significant (p < 0.05) increase in the recruitment of NF-κB p65 to the κB binding site on the IL-6 and IL-8 promotor regions by ChIP (Fig. 7). JQ1 + resulted in non-significant decreases in the recruitment of NF-κB p65 to the κB binding site on the IL-6 (Fig. 7A and B) and IL-8 (Fig. 7C and D) promotor regions to near basal levels in control and PAH HPASMC.

TNFα-stimulation of HPASMC from control and PAH patients resulted in an increase in the recruitment of BRD4 to the IL-6 and IL-8 promoter κB binding sites (Fig. 7). Recruitment was greater in PAH cells compared to controls and higher than that seen in HPMEC. JQ1 + decreased the recruitment of BRD4 to the κB binding site in the IL-6 promotor region (Fig. 7E and F) and in the IL-8 promotor (Fig. 7G and H) to near basal levels but this didn’t reach statistical significance.

## Discussion

We confirm the previously reported upregulation of BRD4 expression in endothelial and smooth muscle cells in PAH lung tissue compared to control lung tissue from patients with normal lung function [18, 26, 32] and extended those studies to also show increased nuclear localisation of BRD2 in PAH cells. TNFα-driven IL-6 protein release from both HPMECs and HPASMCs was greater in PAH cells than from control cells despite greater levels of IL-6 mRNA being induced in cells from control subjects. Similarly, TNFα-induced CXCL8/IL-8 mRNA was much greater in healthy control HPMECs and HPASMCs than in PAH cells although elevated levels of CXCL8/IL-8 protein release was only seen in HPASMCs with similar levels observed in HPMECs. These differences in mRNA expression between healthy control and PAH cells were not due to changes in TNFα-induced NF-κB activation or recruitment of activated NF-κB p65 to the IL-6 and CXCL8/IL-8 promoters as these were similar in both cell types and between subject groups. The BET mimic JQ1 suppressed TNFα-induced IL-6 and CXCL8/IL-8 release and mRNA expression to a similar extent in control and PAH HPMECs and HPASMCs reflecting a similar degree of suppression of TNFα-induced NF-κB p65 and BRD4 recruitment to the IL-6 and CXCL8/IL-8 promoters. However, the IC_50_ for JQ1 suppression of CXCL8/IL-8 release was 3-times higher (~ 300 nM) than that for IL-6 release (~ 100 nM) in HPMECs whereas the IC_50_ for IL-6 suppression in HPASMCs was double that for CXCL8/IL-8 suppression (~ 200 vs ~ 100 nM) in HPASMCs.

These results could be linked to dysregulated acetylation in PAH. Previously we have reported that the HAT/HDAC ratio is altered in IPAH patients [31] and here we now demonstrate higher levels of nuclear BRD4 expression in PAH and that PAH smooth muscle cells appear to express more BRD4 than diseased endothelial cells.

Our results show that PAH cells secrete greater levels of IL-6 and CXCL8/IL-8 than cells from healthy control subjects in response to TNFα despite control subjects generating greater levels of mRNA. This suggests that there is dysregulation of post-transcriptional control of the synthesis and release of these proteins in PAH cells. Translational regulation of mRNA transcripts involves 3 major stages: initiation, elongation and termination [33]. RNA binding proteins bind to the 3'-untranslated regions (3'-UTRs) of mRNA to modulate mRNA stability in response to cellular stressors including hypoxia but may also act as translational repressors. Interestingly, the expression of the RNA binding proteins quaking (QKI) and cold-inducible RNA-binding protein (CIRP) are altered in PAH and affect cellular function [34, 35]. Overall, JQ1 has marked anti-inflammatory effects in both cell types studied irrespective of any mechanistic differences regulating IL-6 and CXCL8/IL8 transcription and secretion.

TNFα-stimulated inflammatory responses are associated with activation of the NF-κB pathway [36]. Upon activation NF-κB p65 moves to the nucleus where it forms a transcriptional activator complex with the promotor regions of inflammatory genes [37]. For maximal activity post-translational modifications such as phosphorylation or acetylation can occur which enhances DNA binding and transcriptional activity. Acetylation of p65 at Lysine-310 (K310) is required for optimal NF-κB transcriptional activity [38]. Acetylated p65, as well as acetylated histones, provides an additional binding site for BRD4 and this interaction is thought to be an important target for BET inhibitors to suppress inflammation [39]. In this study we demonstrated that NF-κB was activated in vascular cells from PAH patients and controls, translocating into the nucleus as early as 30 min after TNFα stimulation. We also showed that TNFα increases the recruitment of p65 and BRD4 to the IL-6 and IL-8 promotors. The bromodomain inhibitor JQ1 decreased recruitment of both p65 and BRD4 to the IL-6 and IL-8 promoters and reduced IL-6 and CXCL8 protein release from vascular cells but had no effect on TNFα-stimulated nuclear translocation of NF-κB p65. These results are in accordance with other studies which indicate BET inhibitors can modulate the transcription of NF-κB target genes in macrophages [23], renal tubular epithelial cells [40], tumour cells [41, 42] and rheumatoid fibroblast‐like synoviocytes [43].

In our studies in human primary pulmonary vascular cells, JQ1 had no effect on TNFα-stimulated NF-κB nuclear translocation, however some groups have shown effects of BET inhibition on NF-κB activation as detected by TransAm assays. In TNFα-treated HUVEC, BET inhibition using JQ1 or siRNA attenuated IKK-mediated activation of NF-κB pathway and decreased activation of p38 and JNK MAPKs [44]. The authors also noted that p38 and JNK inhibitors also blocked TNFα-stimulated NF-κB activation. In rheumatoid fibroblast-like-synoviocytes (RA FLSs) BET inhibition with JQ1 or Brd shRNA decreased TNFα-induced NF-κB–dependent transcription of a luciferase reporter gene and NF-κB target genes [43] following attenuation of TNFα-induced phosphorylation of IKKβ and IκBα, and translocation of nuclear NF-κB. The authors suggested that nuclear BET proteins could regulate the NF-κB pathway through changes in early cytoplasmic IKK signaling events. BET inhibition also prevented the cytoplasmic phosphorylation of the p38 MAPK pathway in TNFα-stimulated RA FLSs. Whilst in diffuse large B-cell lymphomas BET inhibition prevented oncogenic IKK activity [45]. These findings indicate that in some cell types BET proteins may affect cytoplasmic signaling through an unknown mechanism(s). It would be interesting to study the cytoplasmic BRD2 protein interactome in PAH pulmonary vascular cells compared to cells from non-PAH control subjects.

We were unable to show any effects of JQ1 on NF-κB nuclear translocation in the time frame studied and we did not explore the effect of JQ1 on the MAPK pathways. In future studies it would be interesting to explore in more detail the mechanisms by which BET proteins could modulate cytoplasmic IKK activity and MAPKs. This may also provide a role for the enhanced cytoplasmic levels of BRD2 seen in PAH pulmonary vessels reported here.

Earlier studies have shown that BRD2 co-operates with BRD4 to enable RNA polymerase 2 recruitment and run through coding region [46]. The combination of genome-wide transcriptomics and epigenetics of BRD2 and BRD4 function in fibroblast-like synovial cells revealed a close co-operativity between BRD2 and BRD4 actions particularly at a super enhancer that controlled the expression of IL-6 and IL-8 [47]. In addition, BRD2 and BRD4 bind to distinct regions of target genes [47] and have distinct protein–protein interactomes that contribute to the JQ1-induced rewiring of the cellular transcriptome [46].

JQ1 inhibited IL-1β and TNFα-induced expression of inflammatory genes by attenuating the chromatin access for key inflammatory transcription factors AP-1 and NF-κB and by reducing inflammatory pathways controlled by these proteins and by the p38 MAPK pathway [47]. BRD2 binding was more affected than BRD4 binding by JQ1 with a predominant effect on genes controlled by joint BRD2/BRD4 complexes which results in enhanced RNAP2 pausing at promoter sites leading to suppressed mRNA expression [48].

In support of the concept that BRD2 and BRD4 may have distinct roles in specific cell types, the BRD4-selective BET mimic AZD5153, unlike the pan-selective JQ1, has differential effects on natural killer cell inflammatory and cytolytic responses [49]. However, RVX208, which preferentially binds to the second bromodomain in BRD2 and BRD4, reversed the inflammatory and proliferative phenotypes of PAH HPMECs and HPASMCs and reversed monocrotaline- and Sugen5416 + hypoxia-induced in vivo models of PAH [26]. In vivo, both nebulised JQ1 and siBRD4 reversed Sugen/hypoxia-induced PAH with improvements in right heart function and pulmonary pressures reported [18]. Additionally, JQ1 was able to increase mitochondrial spare respiratory capacity and decrease membrane potential to restore a healthy phenotype in PAH-PASMC. Elevated BRD4 expression in PAH has been linked to down-regulation of miR-204. BET inhibition by JQ1 or siBRD4 resulted in upregulation of miR-204. BRD4 inhibition decreased NFATc2 and Bcl-2 mRNA levels and Survivin protein expression with an increase in the cell cycle inhibitor p21 resulting in decreased proliferation and increased apoptosis [18]. In these experiments the authors report a greater effect with JQ1 compared to the specific siBRD4 which is not surprising as JQ1 is a pan-BET selective bromodomain inhibitor and may be having synergistic inhibitory effects on the other BRD proteins known to be involved in PAH. As previously discussed BRD4 and BRD2 are involved in inflammation via activation of NF-κB resulting in the release of proinflammatory cytokines which are capable of activating the STAT3 signaling pathway [50, 51] and also causing DNA damage and subsequent down-regulation of miR-204 [18]. Thus BRDs may play a role in initiation of PAH and also sustaining the inflammatory, proproliferative and antiapoptotic phenotype. In an in vivo model of chronic hypoxia together with pulmonary inflammation, I-BET151 restored hemodynamic parameters to levels of control animals and partially corrected right ventricle hypertrophy [21]. These results could be due to IBET151 changing the acetylation profile of HIF-2α and its target genes. These results suggest that BRD inhibition may also be relevant for COPD related PH and other group 3 PH patients [21].

The increased expression of BRD2 and BRD4 in PAH pulmonary vascular cells may reflect an impact of inflammation since IL-6 can enhance control human coronary artery smooth muscle cell expression of BRD4 to levels seen in cells from PAH subjects [32]. Together with results from JQ1 presented here and in similar ex vivo and in vivo models of PAH it highlights the importance of both BRD2 and BRD4 in controlling the function of PAH.

There are limitations to this study, the main one being the small number (n = 4 per group) of subjects studied. Experiments were performed twice per subject where possible, one at a low passage number and again at a higher passage to rule out any effects of passage number on results. The diseased cells were isolated from tissue obtained at late-stage disease so perhaps do not reflect early stages of disease progression. We only investigated IL-6 and IL-8 as NF-κB activated target genes but many more are known and should be investigated. Future studies should use RNA-sequencing (seq) analysis, possibly together with p65 and/or BRD2/4 ChIP-seq or ATAC-seq analysis, to determine widespread differential gene effects resulting from the altered PAH acetylome in both cell types. Only pharmacological inhibition of BET proteins was performed. JQ1 is not selective for BRD4 and effects other BRDs, therefore experiments using specific siRNA or more selective BRD-specific BET mimics need to be performed. In mitigation, we show that both BRD2 and BRD4 had enhanced nuclear expression in pulmonary vascular endothelial and smooth muscle cells in PAH lung tissue and so both may be playing a role in the molecular phenotype of PAH.

## Conclusion

In summary, our data shows that JQ1 can suppress TNFα-stimulated inflammation by disrupting the recruitment of p65 and BRD4 to IL-6 and IL-8 promoters. JQ1 had a greater effect on IL-6 in HPMEC and CXCL8/IL-8 in HPASMC which suggests distinct cell-specific regulatory control of these genes. This was further suggested by the elevated mRNA expression in healthy control samples but enhanced protein secretion in PAH-derived samples. The data support further development of BET mimics as novel anti-inflammatory agents for the treatment of PAH.

## Supplementary Information

**Additional file 1: Figure S1.** TNFα concentration and time course for release of IL-6 and CXCL8 protein from HPMEC. Control (lefthand panels) and PAH (righthand panels) HPMEC were treated with TNFα for 0-24hrs. Supernatants were collected and levels of IL-6 (**A** and **B**) and CXCL8 (**C** and **D**) determined by ELISA. Data, N=8 (4 Donors at 2 different passages) are shown as mean ± SEM. Statistical comparisons were made using Kruskal-Wallis one-way ANOVA with Dunns post-test. *p<0.05, ** p<0.01, *** p<0.001 when compared to Time 0hr.

**Additional file 2: Figure S2.** TNFα time course to determine optimum induction of IL-6 and -8 mRNA in HPMEC. HPMECs from control (lefthand panels) and PAH (righthand panels) patients were treated with media or TNFα (10ng/ml) for 0–24hrs. Cells were collected, RNA extracted, after which cDNA synthesis and RT-PCR was performed for IL-6 (**A & B**) and IL-8 (**C & D)**. Data are shown as mean ± SEM of N=8 (4 Donors at different 2 passages) experiments. Statistical comparisons were made using Kruskal-Wallis one-way ANOVA with Dunns post-test. *p<0.05, ** p<0.01, *** p<0.001 when comparing between groups and #p<0.05, ## p<0.01, ### p<0.001 when comparing within group to Time 0hr.

**Additional file 3: Figure S3.** TNFα concentration and time course for release of IL-6 and CXCL8 protein from HPASMC. Control (lefthand panels) and PAH (righthand panels) HPASMCs were treated with TNFα for 0–24hrs. Supernatants were collected and levels of IL-6 (**A** and **B**) and CXCL8 (**C** and **D**) determined by ELISA. Data, N=8 (4 Donors at 2 different passages) is shown as mean ± SEM. Statistical comparisons were made using Kruskal-Wallis one-way ANOVA with Dunns post-test. *p<0.05, ** p<0.01, *** p<0.001 when compared to Time 0hr.

**Additional file 4: Figure S4.** TNFα time course to determine optimum time for IL-6 and -8 mRNA induction in HPASMC. HPASMCs from control (lefthand panels) and PAH (righthand panels) patients were treated with media or TNFα (10ng/ml) for 0–24hrs. Cells were collected, RNA extracted, after which cDNA synthesis and RT-PCR was performed for IL-6 (**A** and **B**) and IL-8 (**C** and **D)**. Data, N=8 (4 Donors at 2 different passages) are shown as mean ± SEM. Statistical comparisons were made using Kruskal-Wallis one-way ANOVA with Dunns post-test. *p<0.05, ** p<0.01, *** p<0.001 when comparing between groups and #p<0.05, ## p<0.01, ### p<0.001 when comparing within group to Time 0hr.

## Data Availability

The datasets used and/or analysed during the current study are available from the corresponding author on reasonable request.

## Abbreviations

BET: Bromodomain and extraterminal protein family
BRD2: Bromodomain-containing protein 2
BRD4: Bromodomain containing protein 4
BSA: Bovine serum albumin
ChIP: Chromatin immunoprecipitation
EDTA: Ethylenediaminetetraacetic acid
FCS: Foetal calf serum
HAT: Histone acetyltransferases
HDAC: Histone deacetylases
HPASMC: Human pulmonary artery smooth muscle cells
HPMEC: Human pulmonary microvascular endothelial cells
IKK: Inhibitor of nuclear factor-κB kinase
IL-6: Interleukin 6
IL-8/CXCL8: Interleukin 8
LPS: Lipopolysaccharides
MAPK: Mitogen-activated protein kinase
NF-κB: Nuclear factor kappa-light-chain-enhancer of activated B cells
PAH: Pulmonary arterial hypertension
TNFα: Tumour necrosis factor α
