# Supplementary figures and images for: Differential responses of pulmonary vascular cells from PAH patients and controls to TNFα and the effect of the BET inhibitor JQ1
Source: Respir Res. 2023 Jul 29;24:193. doi: 10.1186/s12931-023-02499-y (PMC10386603; doi:10.1186/s12931-023-02499-y)

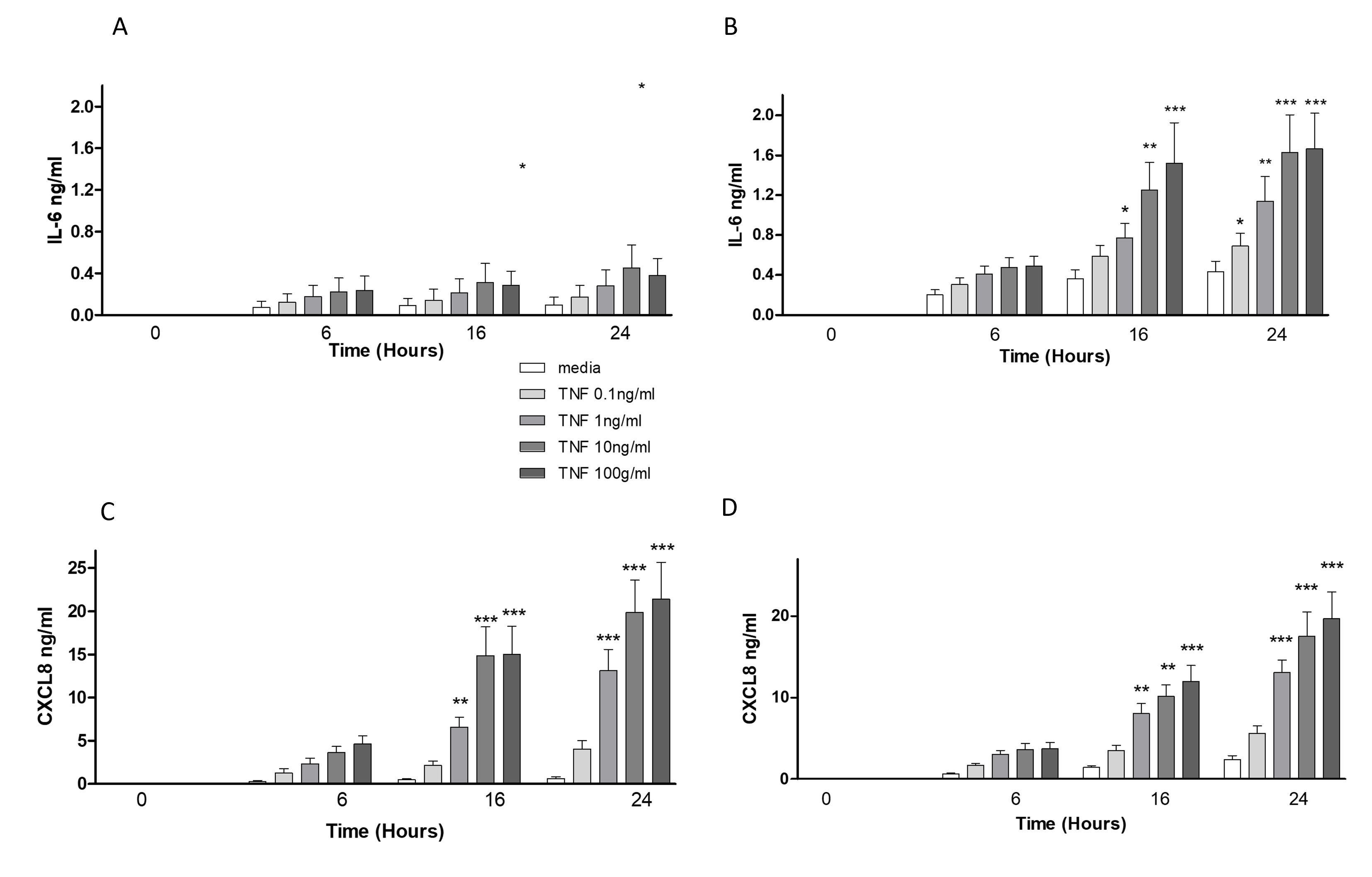

Supplement: Supplementary file 1 — Additional file 1: Figure S1. TNFα concentration and time course for release of IL-6 and CXCL8 protein from HPMEC. Control (lefthand panels) and PAH (righthand panels) HPMEC were treated with TNFα for 0-24hrs. Supernatants were collected and levels of IL-6 (A and B) and CXCL8 (C and D) determined by ELISA. Data, N=8 (4 Donors at 2 different passages) are shown as mean ± SEM. Statistical comparisons were made using Kruskal-Wallis one-way ANOVA with Dunns post-test. *p<0.05, ** p<0.01, *** p<0.001 when compared to Time 0hr. [file 12931_2023_2499_MOESM1_ESM.tif]

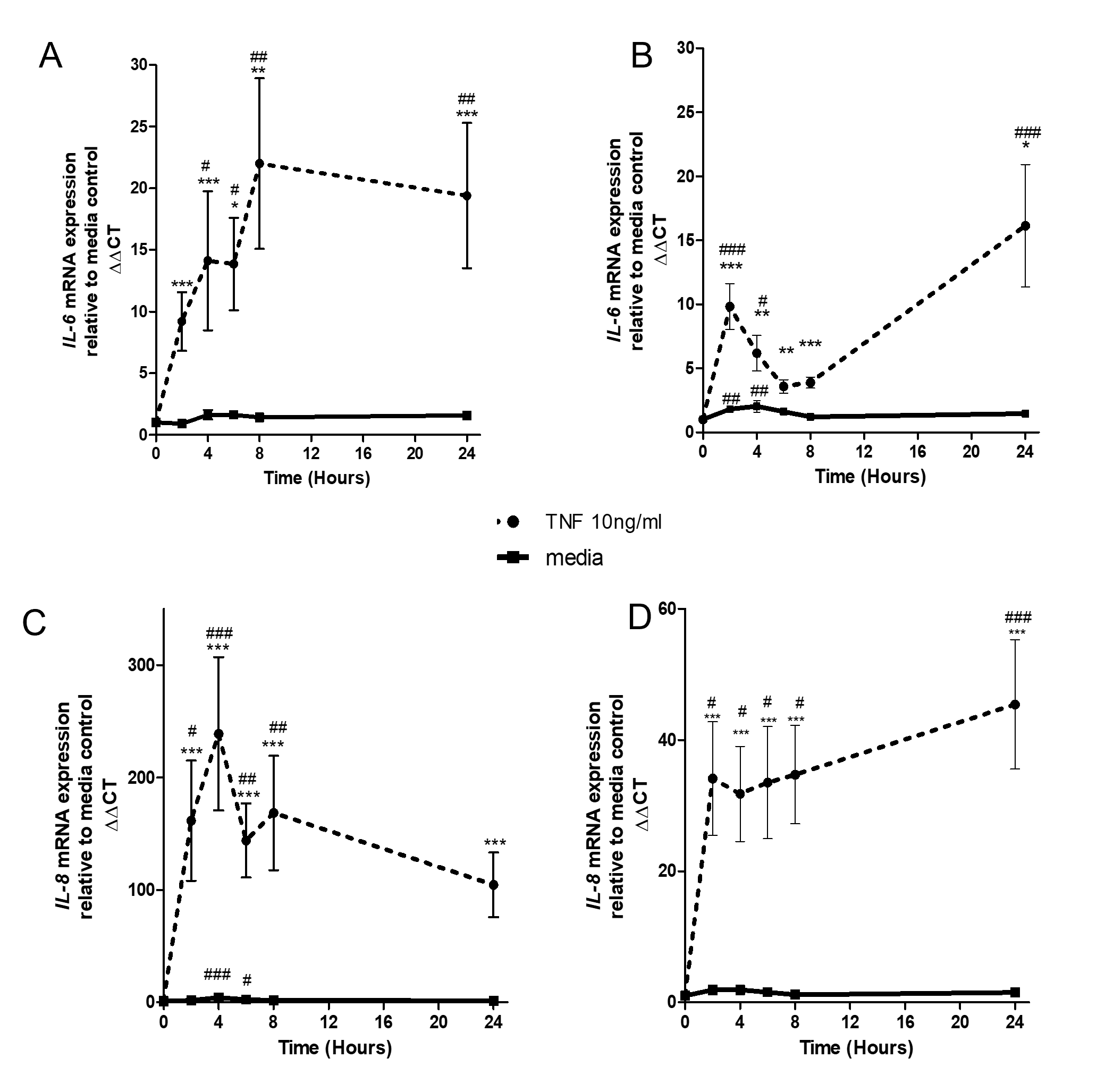

Supplement: Supplementary file 2 — Additional file 2: Figure S2. TNFα time course to determine optimum induction of IL-6 and -8 mRNA in HPMEC. HPMECs from control (lefthand panels) and PAH (righthand panels) patients were treated with media or TNFα (10ng/ml) for 0–24hrs. Cells were collected, RNA extracted, after which cDNA synthesis and RT-PCR was performed for IL-6 (A & B) and IL-8 (C & D). Data are shown as mean ± SEM of N=8 (4 Donors at different 2 passages) experiments. Statistical comparisons were made using Kruskal-Wallis one-way ANOVA with Dunns post-test. *p<0.05, ** p<0.01, *** p<0.001 when comparing between groups and #p<0.05, ## p<0.01, ### p<0.001 when comparing within group to Time 0hr. [file 12931_2023_2499_MOESM2_ESM.tif]

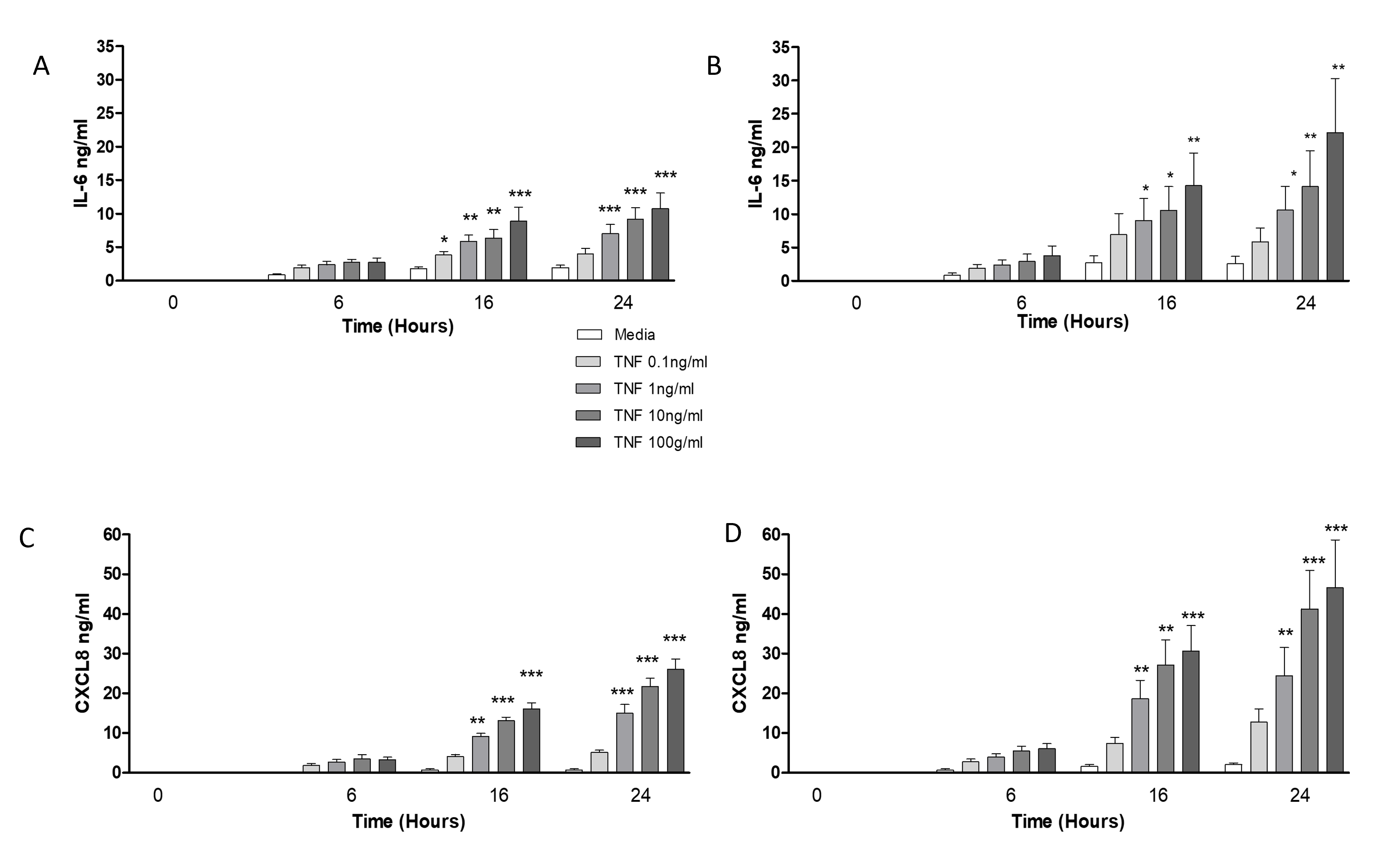

Supplement: Supplementary file 3 — Additional file 3: Figure S3. TNFα concentration and time course for release of IL-6 and CXCL8 protein from HPASMC. Control (lefthand panels) and PAH (righthand panels) HPASMCs were treated with TNFα for 0–24hrs. Supernatants were collected and levels of IL-6 (A and B) and CXCL8 (C and D) determined by ELISA. Data, N=8 (4 Donors at 2 different passages) is shown as mean ± SEM. Statistical comparisons were made using Kruskal-Wallis one-way ANOVA with Dunns post-test. *p<0.05, ** p<0.01, *** p<0.001 when compared to Time 0hr. [file 12931_2023_2499_MOESM3_ESM.tif]

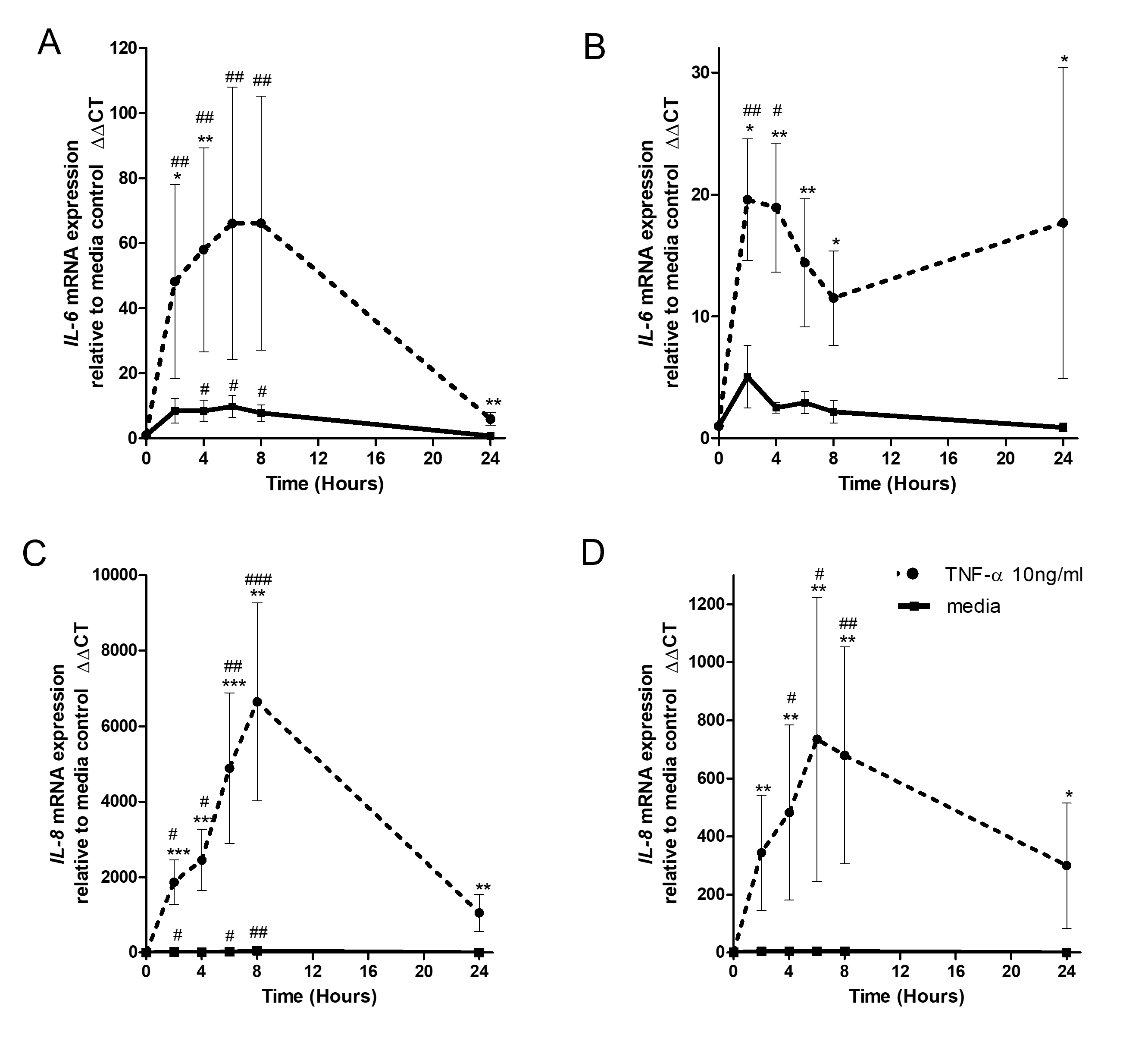

Supplement: Supplementary file 4 — Additional file 4: Figure S4. TNFα time course to determine optimum time for IL-6 and -8 mRNA induction in HPASMC. HPASMCs from control (lefthand panels) and PAH (righthand panels) patients were treated with media or TNFα (10ng/ml) for 0–24hrs. Cells were collected, RNA extracted, after which cDNA synthesis and RT-PCR was performed for IL-6 (A and B) and IL-8 (C and D). Data, N=8 (4 Donors at 2 different passages) are shown as mean ± SEM. Statistical comparisons were made using Kruskal-Wallis one-way ANOVA with Dunns post-test. *p<0.05, ** p<0.01, *** p<0.001 when comparing between groups and #p<0.05, ## p<0.01, ### p<0.001 when comparing within group to Time 0hr. [file 12931_2023_2499_MOESM4_ESM.tif]
